# Detailed composition of wax esters in mouse sebum and the involvement of FAR2 and AWAT2

**DOI:** 10.1016/j.isci.2026.114836

**Published:** 2026-01-29

**Authors:** Karin Kuribayashi, Keisuke Jojima, Moe Yamamoto, Mirei Takeda, Akio Kihara

**Affiliations:** 1 Faculty of Pharmaceutical Sciences, Hokkaido University, Sapporo, Hokkaido 060-0812, Japan

**Keywords:** Natural sciences, Biological sciences, Biochemistry, Physiology

## Abstract

Wax esters (WEs) are lipids characteristic of sebaceous glands and are classified into wax monoesters (WmEs) and wax diesters (WdiEs). Here, we analyzed WEs in mouse sebum via liquid chromatography-tandem mass spectrometry to characterize their composition and structural diversity. We found that mouse sebum contained type 2α and type 2ω WdiEs and revealed the detailed species composition of these WdiEs together with WmEs. In knockout mice of the acyl-CoA reductase *Far2*, the quantities of WEs with very-long-chain fatty alcohols were reduced relative to wild-type mice. In knockout mice of the acyl-CoA wax alcohol acyltransferase *Awat2*, the quantities of many WmEs and type 2ω WdiEs containing long-chain fatty acids were decreased. Sebum WEs were substantially more diverse than meibum WEs. These findings reveal that mouse sebum contains WEs comprising a variety of fatty acids and fatty alcohols and provide clues to the molecular mechanism of their production.

## Highlights

• Mouse sebum is composed of a variety of wax esters (WEs)
• Mouse sebum contains 2α- and 2ω-type wax diesters
• *Far2* and *Awat2* are involved in the synthesis of specific WE species

## Graphical abstract

**Figure:**
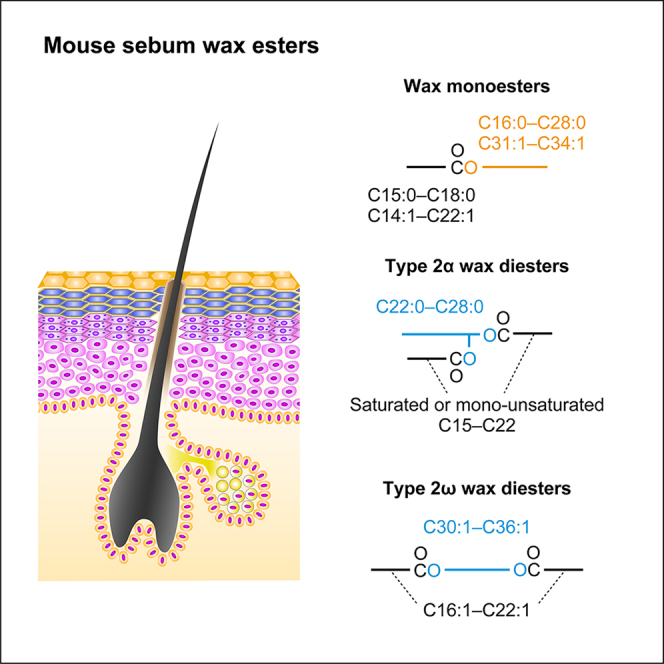

## Introduction

Sebum in the skin plays several roles, including hair and skin lubrication, water repellency, antimicrobial protection, body temperature maintenance, and hair maintenance.^1^^,^^2^ A change in the quantity of sebum or in its composition can cause skin disorders such as acne, dry skin, seborrheic dermatitis, and alopecia.^1^^,^^2^^,^^3^ Sebum is secreted from the sebaceous glands. Most sebaceous glands are associated with hair follicles, and those that are independent of them are called free sebaceous glands.^2^ Free sebaceous glands include the meibomian glands in the eyelids. The lipids secreted from the meibomian glands (meibum lipids) form a lipid layer in the tear film, protecting the cornea and preventing dry eye disease.^4^^,^^5^

**Figure 1:**
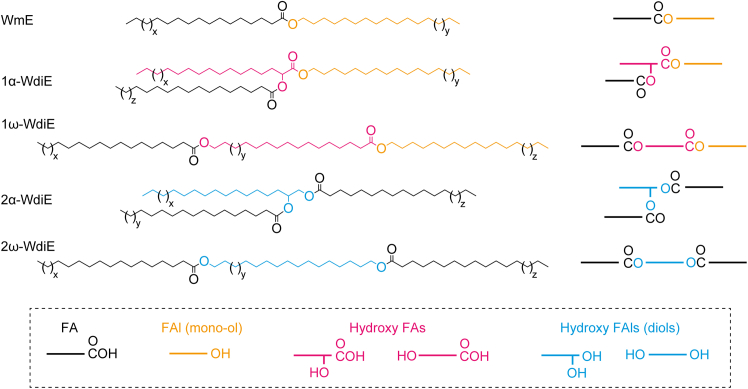
Structures of WEs Detailed and simplified structures of WmE and four types of WdiEs are shown, with the components shown in a dotted box.

Wax esters (WEs) are lipids characteristic of sebum. WEs are classified into wax monoesters (WmEs) and wax diesters (WdiEs). WmEs are composed of a fatty acid (FA) and a fatty alcohol (FAl) (Figure 1). WdiEs are divided into two types. Type 1 WdiEs have a hydroxy FA, with a FAl bound to its carboxyl group and an FA bound to its hydroxyl group. In contrast, type 2 WdiEs have a fatty diol, with each hydroxyl group bound to an FA. WdiEs are further classified into type 1α WdiEs (1α-WdiEs), type 1ω WdiEs (1ω-WdiEs), type 2α WdiEs (2α-WdiEs), and type 2ω WdiEs (2ω-WdiEs), depending on the position of the hydroxyl group (α or ω position) of the hydroxy FA or fatty diol moiety.

Sebum lipids have been analyzed via thin-layer chromatography (TLC) and mass spectrometry (MS).^6^^,^^7^^,^^8^^,^^9^^,^^10^^,^^11^^,^^12^^,^^13^^,^^14^^,^^15^^,^^16^^,^^17^^,^^18^^,^^19^ However, TLC allows only rough discrimination between lipids with different polarities and is poorly quantitative. MS is more quantitative than TLC and can distinguish lipids by their mass-to-charge ratio (*m/z*) after ionization, but it cannot separate lipids with the same *m/z* value. In the analysis of WEs, a classical method—still in use today—involves alkaline hydrolysis to break down WEs into FAs and FAls, followed by the derivatization of these components and analysis using gas chromatography coupled with MS via electron ionization.^6^^,^^7^^,^^8^^,^^9^ In more recent implementations, intact WEs are ionized using atmospheric pressure chemical ionization^10^^,^^12^^,^^19^ or the softer electrospray ionization,^11^^,^^14^^,^^15^^,^^16^^,^^17^^,^^18^ and subsequently analyzed by MS. In such approaches, liquid chromatography (LC) equipped with a reversed-phase column is often employed prior to MS to separate WEs based on hydrophobicity. However, WEs with the same (or very similar) *m/z* values and hydrophobicity cannot be separated even by LC-MS. These include WmEs with the same total carbon chain length but different FA/FAl composition (e.g., C16:1 FA/C26:0 FAl and C18:1 FA/C24:0 FAl). In addition, 1ω-WdiEs and 2ω-WdiEs with the same carbon chain length cannot be separated by LC-MS either.^18^ To separate these, analysis using LC-MS/MS, a combination of LC and tandem mass spectrometry (MS/MS), is necessary. LC-MS/MS allows precursor ions to be dissociated into characteristic product ions according to their structures via collision with an inert gas, making it possible to separate lipids with different structures. In particular, LC-MS/MS in multiple reaction monitoring (MRM) mode shows high specificity, sensitivity, and quantitation by selecting both precursor and product ions based on their respective *m/z* values. We recently established an LC-MS/MS system in MRM mode that can comprehensively measure meibum lipids, including WmEs and WdiEs.^17^^,^^18^ Using this system, we found that mouse meibum lipids contain 2α-WdiEs, 1ω-WdiEs, and 2ω-WdiEs, in addition to WmEs, and revealed their detailed FA/FAl composition.^17^^,^^18^ To date, however, such a method has not been applied to sebum analysis, and the detailed composition of WmEs and the types/compositions of WdiEs have remained unknown.

The WE synthesis pathway includes two reactions: the production of FAl and the formation of an ester bond between an FA and an FAl. FAl production is catalyzed by fatty acyl-CoA reductases (FARs). In mammals, there are two FARs: FAR1 and FAR2.^20^ FAR1 shows high activity toward C16 and C18 of the long-chain (C11–C20) acyl-CoAs, whereas FAR2 prefers very-long-chain (≥C21) acyl-CoAs as substrates.^21^ We generated *Far2* knockout (KO) mice and found that they exhibited dry eye.^21^ In their meibum, almost all WmEs and WdiEs were absent, except for those with C16:0 or C17:0 FAl. Another group reported that *Far2* KO mice exhibited age-dependent alopecia.^16^ However, in that study, only one group of WmE species, in which the sum of the FAl and FA moieties was C39:2, was significantly reduced in the sebum of *Far2* KO mice relative to wild-type (WT) mice (∼30% of WT mice).

Ester bond formation in WEs is catalyzed by acyl-CoA wax alcohol acyltransferases (AWATs). Mammals have two AWATs: AWAT1 and AWAT2.^22^^,^^23^ We generated *Awat2* KO mice and found that they exhibited dry eye.^18^ In their meibum, WmEs and 2ω-WdiEs were almost completely absent, whereas the quantities of 2α-WdiEs were comparable to those in WT mice. At about the same time, another group also reported that *Awat2* KO mice exhibited dry eye and altered meibum lipid composition.^24^ In addition, they reported scaly skin on the tail and ears and partial hair loss on the dorsal neck in the KO mice. However, they did not conduct a lipid analysis of the sebum, leaving the contribution of AWAT2 to WE production in the sebum unknown.

In this study, we comprehensively analyzed WEs in WT mouse sebum via LC-MS/MS in MRM mode. We then applied this method to *Far2* KO mice and *Awat2* KO mice. Through these analyses, we revealed the detailed lipid composition of mouse sebum and the involvement of FAR2 and AWAT2 in WE production.

## Results

### Wax monoester composition in mouse sebum

**Figure 2:**
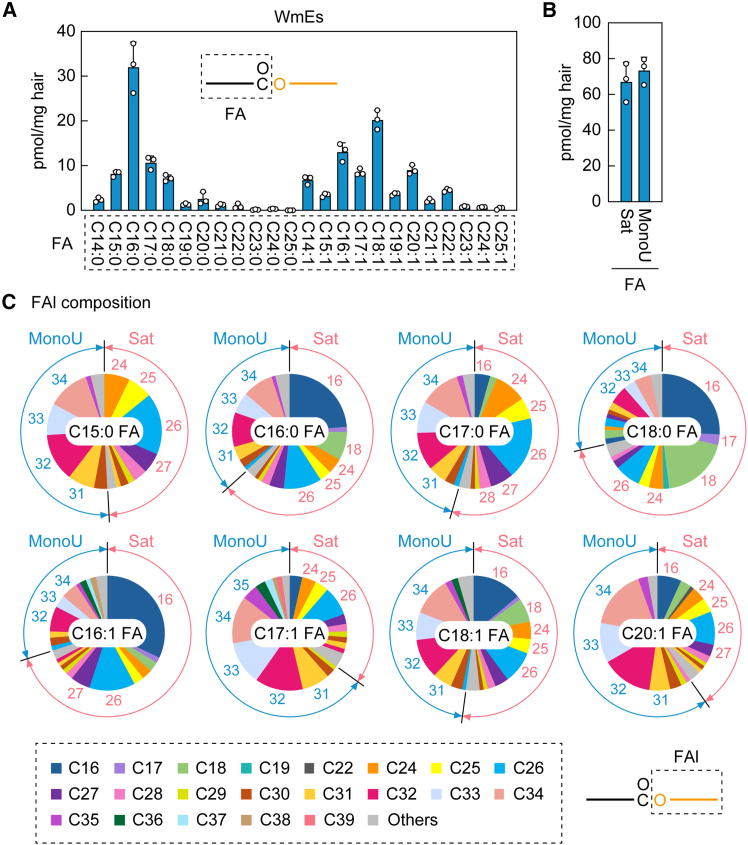
Composition of WmEs in mouse sebum (A–C) Lipids were extracted from the hair of 8-week-old male C57BL/6 mice (*n* = 3), and WmEs were analyzed via LC-MS/MS. Values are the quantities of the WmEs containing the indicated FA moieties (A) and the sum of the quantities of WmEs containing saturated or mono-unsaturated FAs (B). Values shown are means +SD. Each pie chart represents the FAl composition of the WmE containing the indicated FA (C). Numbers in pie charts indicate carbon chain lengths of FAl moieties. The simplified structures of the WmEs with the analyzed moieties (FA or FAl) are shown. Sat, saturated; MonoU, mono-unsaturated.

To reveal the detailed composition of WmEs in mouse sebum, we quantified WmEs in sebum prepared from the hair of WT mice via LC-MS/MS in MRM mode. The parameters were set to measure WmEs containing saturated and mono-unsaturated C14–C25 FAs and C16–C40 FAls (Table S1). The most abundant FA moiety was C16:0, followed by C18:1 and C16:1 (Figure 2A). The total quantity of WmEs containing saturated FAs was similar to that of those containing mono-unsaturated FAs (Figure 2B). WmEs with odd-chain FAs were detected at relatively high levels (Figure 2A). The FAl composition varied among different FA-containing WmEs (Figures 2C and S1A). The chain lengths of the FAl moieties were mainly C16–C28 for saturated FAls and C31–C34 for mono-unsaturated FAls. The most abundant FAl moieties were C16:0 FAl for WmEs containing C16:0 FA, C18:0 FA, C16:1 FA, or C18:1 FA; C26:0 FAl for those containing C15:0 FA or C17:0 FA; C32:1 FAl for those containing C17:1 FA; and C34:1 FAl for those containing C20:1 FA. In summary, we revealed that mouse sebum contains a variety of WmE species.

### Wax diesters composition in mouse sebum

**Figure 3:**
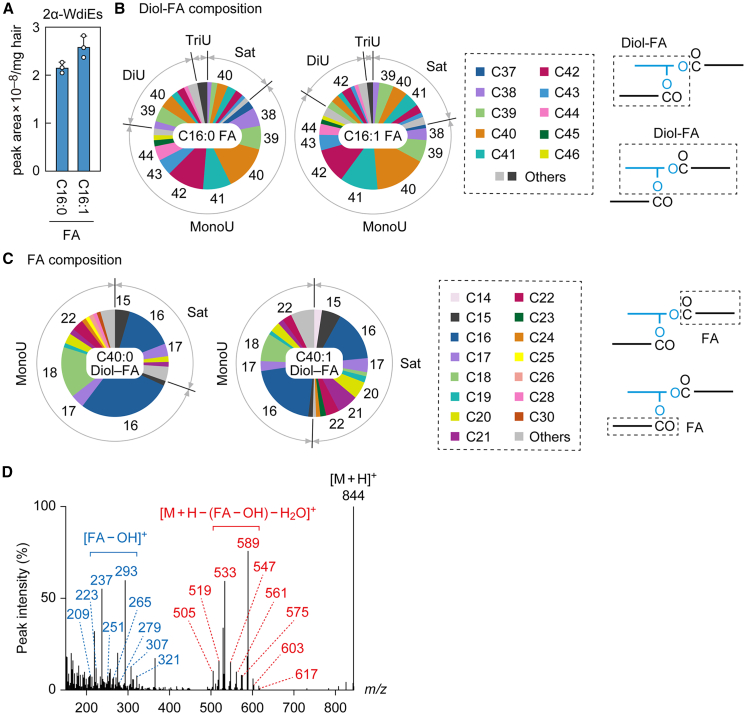
Composition of 2α-WdiEs in mouse sebum (A–D) Lipids were extracted from the hair of 8-week-old male C57BL/6 mice (*n* = 3), and 2α-WdiEs were analyzed via LC-MS/MS in MRM mode (A–C) or product ion scan mode (D). (A–C) Values are the quantities of the lipid species containing the indicated FA moieties (A). Each pie chart represents the diol-FA composition of the 2α-WdiE containing the indicated FA (B) or the FA composition of the 2α-WdiE containing the indicated diol-FA (C). Numbers in pie charts indicate carbon chain lengths of diol-FA (B) or FA (C) moieties. The simplified structures of the 2α-WdiEs with the analyzed moiety (diol-FA conjugate or FA) are shown. Sat, saturated; MonoU, mono-unsaturated; DiU, di-unsaturated; TriU, tri-unsaturated. (D) MS spectrum obtained by product ion scan of the precursor ion with *m/z* 844.

**Figure 4:**
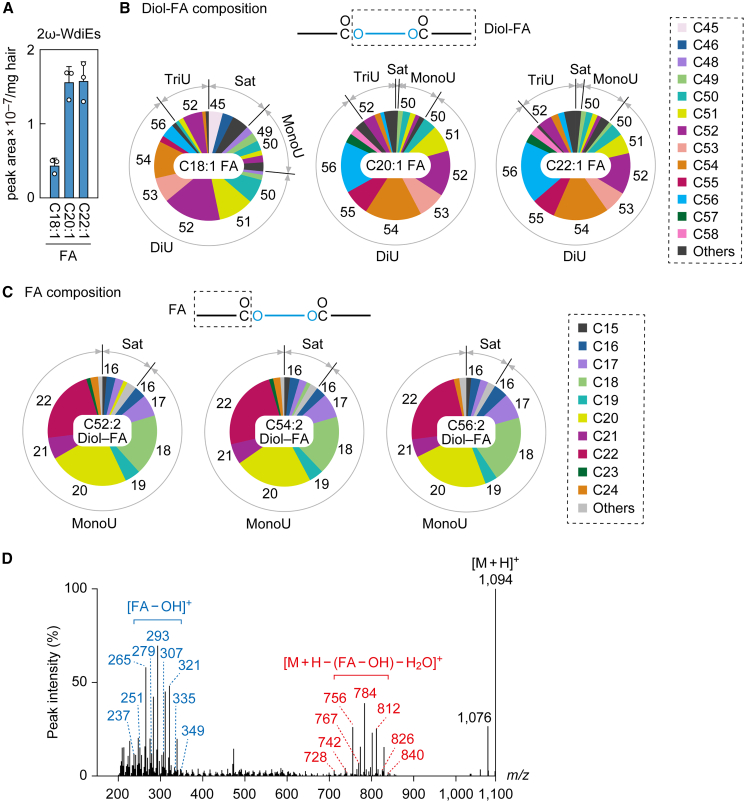
Composition of 2ω-WdiEs in mouse sebum (A–D) Lipids were extracted from the hair of 8-week-old male C57BL/6 mice (*n* = 3), and 2ω-WdiEs were analyzed via LC-MS/MS in MRM mode (A–C) or product ion scan mode (D). (A–C). Values are the quantities of the lipid species containing the indicated FA moieties (A). Values shown are means + SD. Each pie chart represents the diol-FA composition of the 2ω-WdiE containing the indicated FA (B) or the FA composition of the 2ω-WdiE containing the indicated diol-FA (C). Numbers in pie charts indicate carbon chain lengths of diol-FA (B) or FA (C) moieties. The simplified structures of the 2ω-WdiEs with the analyzed moiety (diol-FA conjugate or FA) are shown. Sat, saturated; MonoU, mono-unsaturated; DiU, di-unsaturated; TriU, tri-unsaturated. (D) MS spectrum obtained by product ion scan of the precursor ion with *m/z* 1,094.

Although WdiEs are classified into several types, it was unknown which types of WdiEs exist in mouse sebum. We had previously established an LC-MS/MS system in MRM mode that could distinguish the different types of WdiEs and, using this system, we showed that 1ω-, 2α-, and 2ω-WdiEs exist in mouse meibum.^17^^,^^18^ In this study, we applied this system to mouse sebum and found that WT mice contain 2α- and 2ω-WdiEs (Figures 3 and 4) but at most small quantities of 1ω-WdiEs. This result is consistent with a previous TLC analysis, which found that mouse sebum contains type 2 WdiEs but not type 1 WdiEs.^9^

Structurally, 2α-WdiEs consist of one α-hydroxy (α-OH) FAl (1,α-diol) and two ester-bonded FAs at the 1 and α-positions (Figure 1). Our LC-MS/MS system can identify one FA and the remaining portion (the diol-FA portion; Table S2). First, we measured 2α-WdiEs containing C16:0 or C16:1 FAs and saturated, mono-unsaturated, di-unsaturated, or tri-unsaturated C32–C60 diol-FAs. The total quantity of 2α-WdiEs containing C16:0 FA was comparable to that of those containing C16:1 FA (Figure 3A). Regarding the saturation/unsaturation status of the diol-FA moieties, the most abundant diol-FAs were mono-unsaturated, and substantial quantities of saturated and di-unsaturated diol-FAs were also detected (Figures 3B and S1B). However, only trace quantities of tri-unsaturated diol-FAs were observed. The carbon chain lengths of the diol-FA moieties were mostly C38–C44. In particular, C40:1 in the mono-unsaturated and C40:0 in the saturated diol-FAs were the most abundant. Next, we examined the FA composition of 2α-WdiEs containing C40:0 or C40:1 diol-FA moieties. We found that in both types of 2α-WdiEs, the most abundant FA moiety was C16:1, followed by C16:0 and C18:1 (Figure 3C). The precursor ion of the 2α-WdiE species, consisting of C16:1 FA and C40:1 diol-FA, which represent the most abundant FA and diol-FA moieties, has an *m/z* value of 844. A product ion scan of ions with an *m/z* of 844 revealed two clusters: one corresponding to the FA moiety ([FA – OH]^+^) and the other to the diol-FA moiety ([M + H – (FA – OH) – H_2_O]^+^) (Figure 3D). The former cluster mainly originated from FAs with C14:1–C22:1, and the latter from diol-FAs with C34:1–C42:1. These clusters included *m/z* 237 and 589, corresponding to C16:1 FA and C40:1 diol-FA, respectively.

In 2ω-WdiEs, one ω-OH FAl (1,ω-diol) is ester-bonded with two FAs each at the 1 and ω-positions (Figure 1). Again, our LC-MS/MS system can only identify one FA and the remaining portion (the diol-FA portion; Table S2). We first measured 2ω-WdiEs containing C18:1, C20:1, or C22:1 FAs and saturated, mono-unsaturated, di-unsaturated, or tri-unsaturated C32–C60 diol-FAs. The levels of 2ω-WdiEs containing C20:1 FA or C22:1 FA were similar to each other and higher than those of 2ω-WdiEs containing C18:1 FA (Figure 4A). Regarding the saturation/unsaturation status of the diol-FA moieties, di-unsaturated diol-FAs were more abundant than the other categories (Figures 4B and S1C). The most abundant diol-FA moieties were C52:2, C54:2, and C56:2 in 2ω-WdiEs containing C18:1, C20:1, and C22:1 FAs, respectively. We next examined the FA composition of 2ω-WdiEs containing C52:2, C54:2, or C56:2 diol-FAs. In all cases, 2ω-WdiEs contained predominantly mono-unsaturated FA moieties, with a minor fraction of saturated FAs. These mono-unsaturated FA moieties were primarily in the C16–C22 range, with C20:1 and C22:1 being most abundant, followed by C18:1 (Figure 4C). The precursor ion of 2ω-WdiE, which contains the most abundant FA (C20:1) and diol-FA (C54:2), has an *m/z* of 1,094. A product ion scan of species sharing an *m/z* of 1,094 revealed two clusters: one corresponding to the FA moiety ([FA – OH]^+^) and the other to the diol-FA moiety ([M + H – (FA – OH) – H_2_O]^+^) (Figure 4D). The former cluster mainly originated from FAs with C16:1–C24:1, whereas the latter derived from diol-FAs of C50:2–C58:2. The most intense peaks at *m/z* 293 and 784 corresponded to C20:1 FA and C54:2 diol-FA. Additionally, species sharing the same precursor *m/z* (C18:1 FA–C56:2 diol-FA and C22:1 FA–C52:2 diol-FA) produced secondary major product ions ([FA – OH]^+^ at 265 and 321; [M + H – (FA – OH) – H_2_O]^+^ at 812 and 756, respectively).

### Presence of cholesteryl (*O*-acyl)-ω-OH FAs (Chol-OAHFAs) in sebum

**Figure 5:**
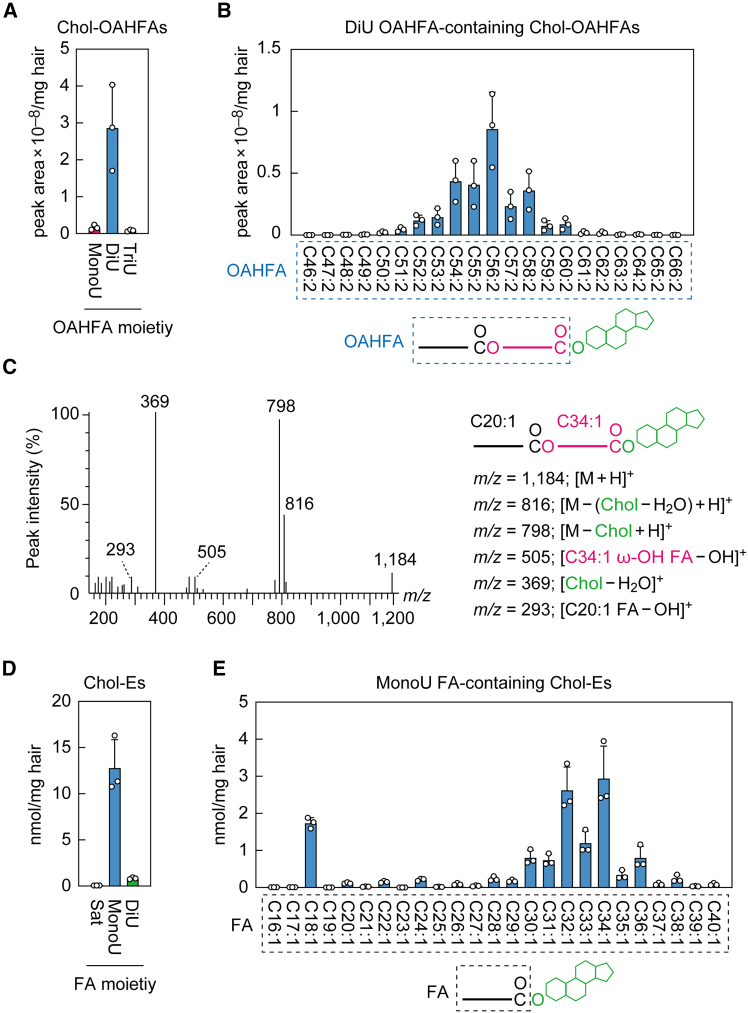
Existence of Chol-OAHFAs and composition of Chol-Es in mouse sebum (A–E) Lipids were extracted from the hair of 8-week-old male C57BL/6 mice (*n* = 3), and Chol-OAHFAs (A–C) and Chol-Es (D and E) were analyzed via LC-MS/MS in MRM mode (A, B, D, and E) or product ion scan mode (C). (A and B) Values are the total quantities of Chol-OAHFAs containing mono-unsaturated, di-unsaturated, or tri-unsaturated OAHFA moieties (A) and the quantities of Chol-OAHFAs containing the indicated di-unsaturated OAHFA moieties (B). Values shown are means +SD. The simplified structure of the Chol-OAHFA with the analyzed OAHFA moiety is shown below the graph (B). (C) MS spectrum obtained by product ion scan of the precursor ion with *m/z* 1,184, corresponding to C54:2 OAHFA-containing Chol-OAHFA. The predicted product ions and the simplified structure of Chol-OAHFA are also shown. (D and E) Values are the total quantities of Chol-Es containing saturated, mono-unsaturated, or di-unsaturated FA moieties (D) and the quantities of Chol-Es containing the indicated mono-unsaturated FA moieties (E). Values shown are means +SD. The simplified structure of the Chol-E with the analyzed FA moiety is shown below the graph (E). Sat, saturated; MonoU, mono-unsaturated; DiU, di-unsaturated; TriU, tri-unsaturated.

Meibum contains a special class of lipids called Chol-OAHFAs,^5^^,^^13^ which consist of a cholesterol and an (*O*-acyl)-ω-OH FA (OAHFA). While their existence in human vernix caseosa was previously reported,^25^ their presence in sebum was unknown until now. To examine this, we performed LC-MS/MS and found that Chol-OAHFAs did exist in mouse sebum (Figures 5A and S1D). Most of their OAHFA moieties were di-unsaturated, with few mono-unsaturated or tri-unsaturated moieties. The most abundant OAHFA moiety was C56:2, followed by C54:2, C55:2, and C58:2 (Figure 5B). Product ion scan analysis of Chol-OAHFAs containing C54:2 OAHFA detected peaks predicted to be derived from C20:1 FA and C34:1 ω-OH FA (Figure 5C).

It is known that mouse sebum contains a large quantity of cholesteryl esters (Chol-Es).^7^ We examined the detailed composition of mouse sebum Chol-Es via LC-MS/MS and found that most of them had mono-unsaturated FAs (Figures 5D and S1E). Of these, the most abundant Chol-E species contained C34:1 FA, followed by C32:1 FA (Figure 5E). In conclusion, both Chol-OAHFAs and Chol-Es in mouse sebum contain mainly mono-unsaturated ω-OH FA and FA moieties longer than C30, respectively.

### Involvement of fatty acyl CoA reductase 2 in sebum wax monoester production

**Figure 6:**
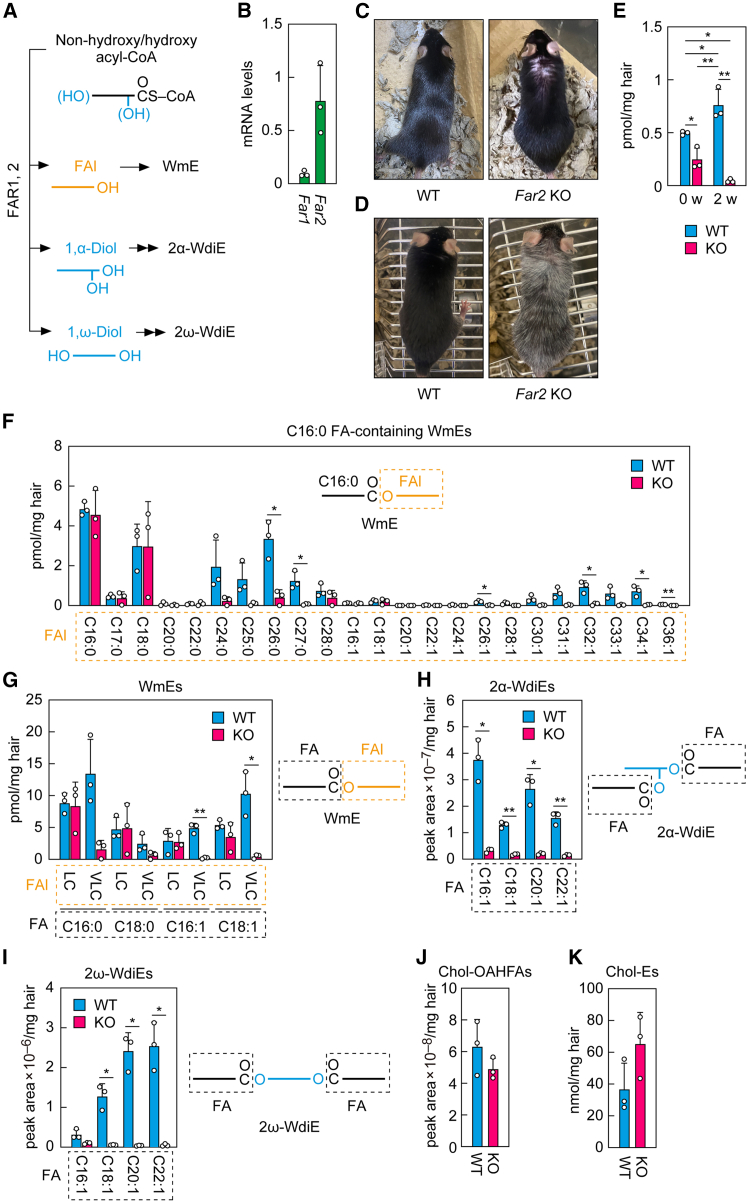
Impaired production of WEs containing very-long-chain FAls in *Far2* KO mouse sebum (A) Conversion of non-hydroxy as well as α- or ω-OH acyl-CoAs to FAls or 1,α/1,ω-diols by FARs, and the metabolic pathways of these products. (B) Total RNAs were prepared from the skin of 8-week-old male C57BL/6 mice (*n* = 3), and the expression levels of *Far1*, *Far2*, and the housekeeping gene *Hprt1* were measured via quantitative real-time RT-PCR. Values presented are mean +SD of the mRNA levels relative to *Hprt1*. (C and D) Photographs of a *Far2* KO male mouse at 6 months of age showing partial hair loss (C) and at 9 months showing whitening hair (D), as well as WT male mice of the same age. (E) WT and *Far2* KO male mice (*n* = 3 each) were housed in the same cage for 2 weeks, after which mice of different genotypes were transferred to separate cages. Lipids were extracted from the hair of the mice 0 and 2 weeks (w) after they were separated, and the WmE with C18:1 FA/C26:0 FAl was quantified via LC-MS/MS. Values presented are mean quantities +SD (∗*p* < 0.05; ∗∗*p* < 0.01; Tukey’s test). (F–K) WT and *Far2* KO male mice (*n* = 3 each) at 5 weeks of age were transferred to separate cages and housed separately for 3 weeks. Lipids were extracted from the hair, and WmEs (F and G), 2α-WdiEs (H), 2ω-WdiEs (I), Chol-OAHFAs (J), and Chol-Es (K) were analyzed via LC-MS/MS. (F and G) Values are the quantities of WmEs containing C16:0 FA and the indicated FAl moieties (F) and those containing the indicated FA and long-chain (LC) or very-long-chain (VLC) FAl moieties (G). Values shown are means +SD (∗*p* < 0.05; ∗∗*p* < 0.01; Welch’s *t* test). The simplified structures of the WmEs with the analyzed moieties (FAl and/or FA) are shown. (H and I) Values are the quantities of 2α-WdiEs (H) and 2ω-WdiEs (I), both containing the indicated FA moieties. Values presented are mean quantities +SD (∗*p* < 0.05; ∗∗*p* < 0.01; Welch’s *t* test). The simplified structures of the 2α-WdiE (H) and 2ω-WdiE (I) with the analyzed FA moieties are shown. (J and K) Values are the means +SD of the total quantities of Chol-OAHFAs (J) and Chol-Es (K).

FARs catalyze the reduction of acyl-CoAs, including non-hydroxy as well as α- or ω-OH acyl-CoAs, to produce FAls or 1,α- and 1,ω-diols. These products are subsequently converted into WmEs or into 2α- or 2ω-WdiEs, respectively (Figure 6A). Of the two FAR isozymes found in mammals, *Far1* is expressed ubiquitously among tissues, while *Far2* shows tissue specificity.^20^ *Far2* is most abundantly expressed in the eyelids (which contain meibomian glands), followed by skin containing sebaceous glands.^20^ We performed quantitative real-time RT-PCR and confirmed that *Far2* was highly expressed in mouse skin, whereas the expression of *Far1* was low (Figure 6B). It has been reported that *Far2* KO mice exhibit age-dependent hair loss.^16^ However, that study reported that only the C39:2 WmE species was statistically significantly reduced in *Far2* KO mice relative to WT mice. In addition, WdiEs were not measured in that study.

To validate the WmE data for *Far2* KO mice and investigate the changes in WdiE composition due to *Far2* deficiency, we performed LC-MS/MS analyses on sebum prepared from WT and *Far2* KO mice. As in the previous study,^16^ we observed the hair-loss phenotype in 19 of 19 *Far2* KO mice (8 males and 11 females) aged four months or older (Figure 6C). In addition, three of the eight male *Far2* KO mice showed whitening of the hair (Figure 6D). Since mice rub against each other, we hypothesized that the composition of their sebum might have been affected by that of other mice living in the same cage. To examine this possibility, we housed WT and *Far2* KO mice together for two weeks, then separated them into different cages, and quantified the levels of the representative WmE with C18:1 FA/C26:0 FAl in the sebum zero and two weeks after the separation. The quantity of that WmE in the KO mice was approximately half that of the WT mice at week 0, but decreased to 7% at week 2 (Figure 6E). In contrast, the quantity of that WmE in WT mice increased 1.5-fold at week 2 relative to week 0. Thus, the quantity of this WmE in KO mouse hair was overestimated at week 0 due to contamination from WT mice, and conversely, that in WT mice was underestimated as a result of transfer to the KO mice. Therefore, mice of different genotypes should be housed in separate cages for at least two weeks to accurately measure the sebum of each genotype. In this study, we used mice that had been kept in separate cages for three weeks for the analyses in the following experiments.

We analyzed the WmEs in sebum from WT and *Far2* KO mice in detail via LC-MS/MS. We first quantified WmEs containing C16:0 FA, the most abundant FA species, and found that the quantities of many species in WmEs containing very-long-chain FAls were greatly reduced in *Far2* KO mice relative to WT mice (Figure 6F), with the sum of these WmEs in *Far2* KO mice at 12% of that in WT mice (Figure 6G). In contrast, the quantities of WmEs containing long-chain C16–C18 FAls were similar in *Far2* KO and WT mice (Figures 6F and 6G). It is therefore likely that these long-chain FAls are produced by FAR1. Similar results were obtained for WmEs containing FAs other than C16:0 FA (Figure 6G).

Next, we investigated the involvement of FAR2 in WdiE production in the sebum. We found that the quantities of both 2α-WdiEs and 2ω-WdiEs, which contain mainly very-long-chain α-OH and ω-OH FAls, respectively (Figures 3B and 4B), were greatly reduced in *Far*2 KO mice relative to WT mice, regardless of their FA moieties (Figures 6H and 6I). However, we observed no significant differences between WT and *Far2* KO mice in the quantities of Chol-OAHFAs and Chol-Es (Figures 6J and 6K), neither of which contains an FAl moiety. These results indicate that FAR2 plays an important role in the production of very-long-chain FAls, which are components of WmEs and WdiEs, not only in meibum as reported previously,^21^ but also in sebum.

### Involvement of acyl-CoA wax alcohol acyltransferase 2 in sebum wax ester production

**Figure 7:**
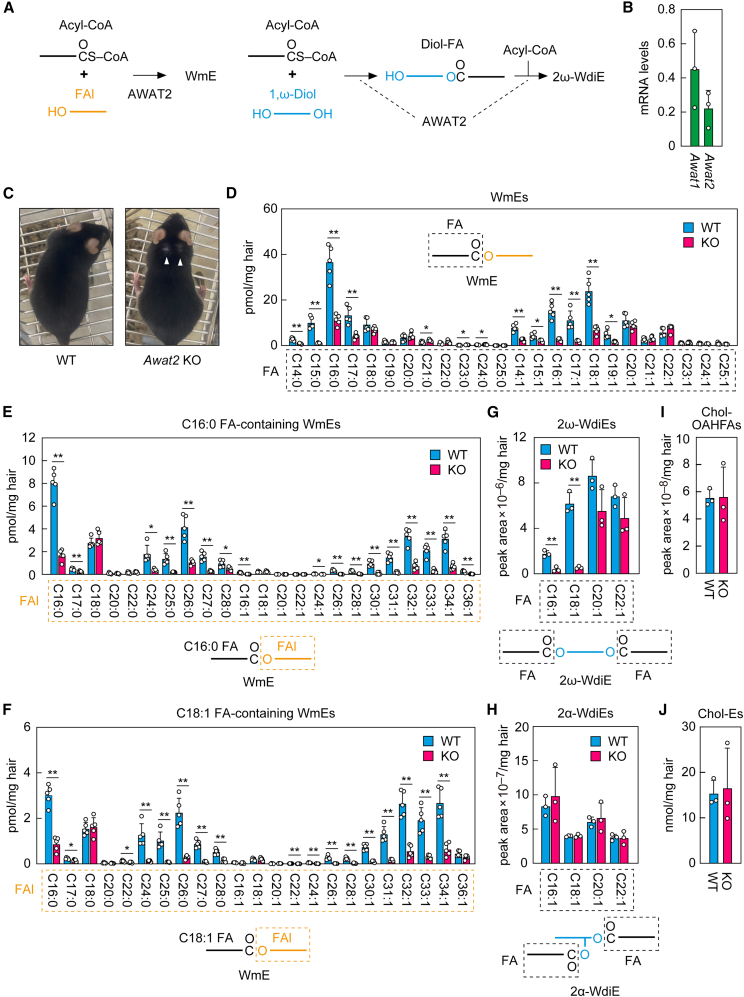
Impaired WmE and 2ω-WdiE production containing long-chain FAs in *Awat2* KO mouse sebum (A) Pathways for the production of WmEs and 2ω-WdiEs by AWAT2. AWAT2 produces WmEs using an acyl-CoA and an FAl as substrates. 2ω-WdiEs are produced through an initial esterification reaction of an acyl-CoA with a 1,ω-diol to form diol-FAs, followed by a second esterification reaction of an acyl-CoA with a diol-FA. AWAT2 catalyzes either or both of these reactions. (B) Total RNAs were prepared from the skin of 8-week-old male C57BL/6 mice (*n* = 3), and the expression levels of *Awat1*, *Awat2*, and the housekeeping gene *Hprt1* were measured via quantitative real-time RT-PCR. Values presented are mean +SD of the mRNA levels relative to *Hprt1*. (C) Photographs of a 9-month-old *Awat2* KO male mouse showing partial hair loss (arrowheads) and a WT male mouse of the same age. (D–J) WT and *Awat2* KO male mice (*n* = 3 each) at 5 weeks of age were transferred to separate cages and housed separately for 3 weeks. Lipids were extracted from the hair, and WmEs (D–F), 2ω-WdiEs (G), 2α-WdiEs (H), Chol-OAHFAs (I), and Chol-Es (J) were analyzed via LC-MS/MS. (D–F) Values are the quantities of the WmEs containing the indicated FA moieties (D) and the WmEs containing C16:0 FA (E) or C16:1 FA (F) and the indicated FAl moieties (E and F). Values shown are means +SD (∗*p* < 0.05; ∗∗*p* < 0.01; Welch’s *t* test). The simplified structures of the WmEs with the analyzed moieties (FA or FAl) are shown. (G and H) Values are the quantities of the 2ω-WdiEs (G) and 2α-WdiEs (H) containing the indicated FA moieties. Values presented are mean quantities +SD (∗∗*p* < 0.01; Welch’s *t* test). The simplified structures of the 2ω-WdiE (G) and 2α-WdiE (H) with the analyzed FA moieties are shown. (I and J) Values are the means +SD of the total quantities of Chol-OAHFAs (I) and Chol-Es (J).

There are two acyl-CoA wax alcohol acyltransferases (AWAT1 and AWAT2) in mammals.^22^^,^^23^ Of these, AWAT2 is involved in the production of WmEs and 2ω-WdiEs in meibum (Figure 7A).^18^ To examine the expression of these acyltransferases in mouse skin, we performed quantitative real-time RT-PCR and found that both were expressed (Figure 7B). Although partial hair loss on the dorsal neck was reported in *Awat2* KO mice,^24^ the quantities of WmEs in sebum were not measured in that study. Therefore, in this study, we analyzed WmEs in the sebum of *Awat2* KO mice. Only a small proportion of the *Awat2* KO mice we generated showed partial hair loss on the dorsal side of the neck (one in six male mice aged three months or older; Figure 7C). In the sebum of *Awat2* KO mice, the quantities of WmEs containing C14–C17 saturated (C14:0–C17:0) FAs and C14–19 mono-unsaturated (C14:1–19:1) FAs of long-chain FAs were reduced relative to WT mice (Figure 7D). In contrast, the quantities of WmEs containing longer FAs (≥C18:0 and ≥C20:1), many of which are classified as very-long-chain FAs, in *Awat2* KO mice were either unchanged or slightly higher than those in WT mice. Of the C16:0 FA-containing WmE species, those containing FAls with a wide range of chain lengths (C16, C17, and ≥C24) were significantly reduced in *Awat2* KO mice relative to WT mice, whereas there was no reduction in WmEs containing C18–C22 FAls (Figure 7E). We obtained a similar result for C18:1 FA-containing WmEs (Figure 7F). These results indicate that although AWAT2 is involved in the production of most of WmEs, there must be another acyltransferase that is involved in the production of WmEs with very-long-chain FAs in particular. AWAT1 was considered as a possible candidate for that acyltransferase. However, WmE levels were substantially increased in *Awat1 Awat2* double KO mice relative to WT mice, ruling out that possibility (Figure S2).

We next examined the effect of *Awat2* KO on WdiE production via LC-MS/MS. We found that the quantities of 2ω-WdiEs containing C16:1 and C18:1 FAs were reduced in *Awat2* KO mice relative to WT mice (C16:1, 22% of WT mice; C18:1, 9%), whereas those of 2ω-WdiEs containing C20:1 and C22:1 FAs were similar to those of WT mice (Figure 7G). This again indicates that AWAT2 is involved in the production of 2ω-WdiEs containing long-chain FAs but not in that of 2ω-WdiEs containing very-long-chain FAs. The quantities of 2α-WdiEs, Chol-OAHFAs, and Chol-Es were similar in WT and *Awat2* KO mice (Figures 7H–7J). These results indicate that AWAT2 is involved in the production of WmEs and 2ω-WdiEs containing long-chain FAs.

### Involvement of acyl-CoA wax alcohol acyltransferases 2 in meibum wax monoester production

**Figure 8:**
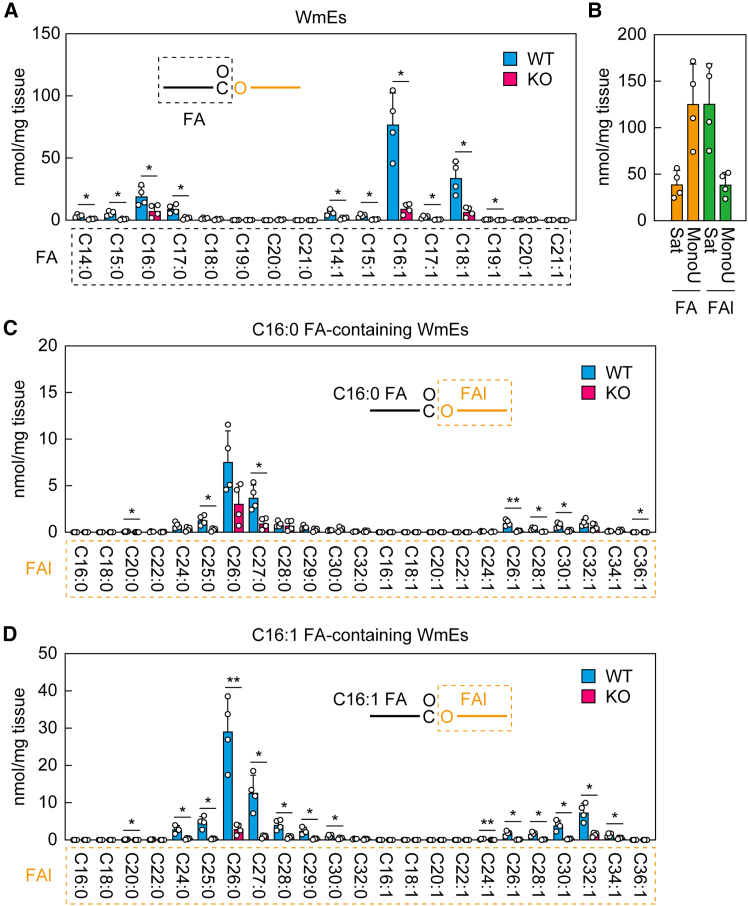
Impaired WmE production in *Awat2* KO mouse meibum (A–D) Lipids were extracted from the eyelids of WT and *Awat2* male KO mice (*n* = 4 each) at 6–10 weeks of age, and WmEs were analyzed via LC-MS/MS. Values are the quantities of the WmEs containing the indicated FA moieties (A), the sum of the quantities of WmEs containing a saturated (Sat) or mono-unsaturated (MonoU) FA or FAl moiety (B), the quantities of WmEs containing C16:0 FA (C) or C16:1 FA (D), and the indicated FAl moieties (C and D). Values shown are means +SD (∗*p* < 0.05; ∗∗*p* < 0.01; Welch’s *t* test). The simplified structures of the WmEs with the analyzed moieties (FA or FAl) are shown.

We previously quantified meibum WmEs containing mono-unsaturated C16–C20 FAs in WT and *Awat2* KO mice,^18^ and in this study, we expanded these measurements to WmEs containing saturated FAs. Unlike sebum WmEs, which contain a wide variety of FA species (mainly C14–C22 saturated and mono-unsaturated FAs) (Figure 2A), only limited FA species were present in the meibum. In addition to the C16:1 and C18:1 FA species that were previously known to be abundant,^18^ we found that the only species with substantial quantities in the meibum were C16:0 and C17:0 FAs, although they were less abundant than the C16:1 and C18:1 FA species (Figure 8A). The total quantity of WmEs containing saturated FAs was approximately one-third of that containing mono-unsaturated FAs (Figure 8B). In *Awat2* KO mice, all these major WmE species were greatly reduced relative to WT mice (Figure 8A).

Among the FAl moieties of the WmEs in WT mouse meibum, the quantity of saturated FAls was approximately triple that of mono-unsaturated FAls (Figure 8B). The FAl composition of the meibum was also less diverse than that of the sebum. Among the WmEs containing C16:0 FA, the chain-lengths of saturated FAls were mainly C24–C28, with C26:0 being the most abundant, followed by C27:0, and those of mono-unsaturated FAls were mainly C26–C32 (Figure 8C). We observed a similar FAl composition for the WmEs containing C16:1 FA (Figure 8D). In *Awat2* KO mice, almost all WmE species were reduced relative to WT mice, regardless of their FAl moiety (Figures 8C and 8D). In summary, meibum WmEs were less diverse than sebum WmEs, and most meibum WmE species were reduced by *Awat2* KO.

## Discussion

In this study, we performed comprehensive analyses of WEs in mouse sebum and revealed the detailed FA and FAl composition of WmEs (Figure 2) and the presence of 2α-WdiEs and 2ω-WdiEs and their detailed composition (Figures 3 and 4). These results indicate the great diversity of sebum WEs in terms of both their FA and FAl portions. We also found that the sebum contains Chol-OAHFAs (Figure 5), and we revealed the contribution of FAR2 and AWAT2 to sebum WE production (Figures 6 and 7).

**Figure 9:**
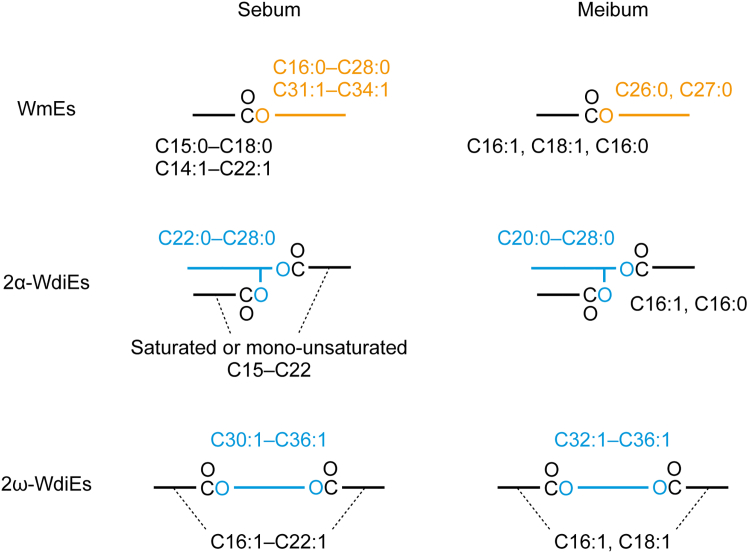
Differences in the constituents of WmEs, 2α-WdiEs, and 2ω-WdiEs in sebum and meibum The predominant chain lengths and degree of unsaturation of the FA, FAl, and/or diol moieties constituting WmEs, 2α-WdiEs, and 2ω-WdiEs in mouse sebum and meibum.

WEs are lipids that are characteristic of sebaceous glands. We observed some differences between the WEs in mouse sebum and meibum (Figure 9). For WmEs, we found that the FA portion of the sebum WmEs contained many types of FAs, including saturated and mono-unsaturated FAs ranging from long-chain to very-long-chain (mainly C14–C22; Figure 2A). Of these, C16:0 FA was the most abundant, followed by C18:1, C16:1, C17:0, and C20:1 FAs. In contrast, the FA moieties in mouse meibum WmEs were less diverse, consisting mainly of C16:1, C18:1, and C16:0 FAs (Figure 8A).^18^ Although the exact reason for this difference in diversity is unclear, it is possible that the FA composition of the sebaceous and meibomian glands differed. The FAl moieties of WmEs were also more diverse in the sebum than in the meibum. In the sebum WmEs, FAls were mainly saturated C16–C28 (C16:0–C28:0) and mono-unsaturated C31–C34 (C31:1–C34:1), and the FAl composition differed substantially among WmEs with different FAs (Figure 2C). In contrast, in the meibum WmEs, there were only a few predominant FAl species (C26:0 and C27:0), and the FAl composition was similar among WmEs with different FAs (Figures 8C and 8D).

The sebum 2α-WdiEs contained mainly saturated and mono-unsaturated C15–22 FAs (Figure 3C). In contrast, the majority of the FA moieties in the 2α-WdiEs in meibum were C16:1 and C16:0 FAs.^18^ The diol-FA moiety was predominantly C38:1–C44:1 (Figure 3B); if it is assumed that the FA portion is C16:1, the most abundant FA, then the diol portion would range from C22:0 to C28:0. This diol composition is similar to that of meibum 2α-WdiE (C20:0–C28:0; Figure 9).^18^ In sebum 2ω-WdiEs, the most abundant diol-FA moiety was C52:2–C56:2 (Figure 4B). Since the FA moiety predominantly contained C20:1 and C22:1 (Figure 4C), the diol portion of C52:2–C56:2 diol-FA moieties is predicted to range from C30:1 to C36:1 (Figure 9). In mouse meibum, the most abundant FA moiety in 2ω-WdiEs was C16:1 FA, followed by C18:1 FA,^18^ which differs from sebum 2ω-WdiEs. In contrast, the diol portion of sebum 2ω-WdiEs (C30:1–C36:1) was similar to that of meibum (C32:1–C36:1).^18^ Thus, although the FA composition of the WdiEs differs between sebum and meibum, the diol composition is similar (Figure 9).

The WEs in the sebum were therefore more diverse than those in the meibum. In addition, the sebum WmEs contained similar levels of mono-unsaturated and saturated FAls (Figure 2C), whereas most of the FAls in the meibum WmEs were saturated (Figure 8). The WE composition in each type of sebaceous gland may be suited to the environmental conditions in which it works, particularly the temperature. Meibum lipids are located in the outermost layer of tear film and are maintained at approximately 32°C, the temperature of the cornea.^26^^,^^27^^,^^28^ At that temperature, meibum lipids are in a liquid crystalline phase.^29^ In contrast, the sebum that covers the hair and skin is affected by the outside environment and is exposed to a wide range of temperatures. In general, the range of melting points and phase-transition temperatures of a mixture becomes wider as the mixture becomes more diverse. We speculate that the WE diversity in sebum is necessary for adaptation to a wide range of environmental temperatures. In addition, it is possible that the relatively high content of mono-unsaturated FAls in sebum WmEs has the effect of lowering the melting point so that the sebum does not solidify even when exposed to low temperatures.

A previous report on *Far2* KO mice showed that *Far2* deficiency did not substantially affect WmE composition, with only the C39:2 species being statistically significantly reduced relative to WT mice (to approximately 30%).^16^ Considering the FA and FAl composition of the WmEs that we revealed in this study (Figure 2), that finding suggests that most of the C39:2 WmEs should be either C18:1 FA/C21:1 FAl or C16:1 FA/C23:1 FAl species, both of which are minor WmE species. In contrast to that report, here we found that the quantities of many WmEs with very-long-chain FAl moieties were substantially reduced in *Far2* KO mice relative to WT mice (Figure 6F). This discrepancy may be mainly due to the housing conditions of the mice. We showed that sebum composition is influenced by contact with other mice in the same cage and that housing mice of different genotypes in separate cages for at least two weeks is necessary for the accurate measurement of the sebum (Figure 6E). Unlike WmEs containing very-long-chain FAls, the quantities of WmEs containing long-chain FAls were not reduced in *Far2* KO mice relative to WT mice (Figures 6F and 6G). We previously reported that FAR1 and FAR2 are responsible for the production of long-chain and very-long-chain FAls, respectively, via cell-based assays.^21^ Therefore, it is highly likely that the long-chain FAl moieties are produced by FAR1. In *Far2* KO mice, quantities of 2α-WdiEs and 2ω-WdiEs, both of which mainly contain very-long-chain α-OH/ω-OH FAls (diols), were significantly reduced relative to WT mice (Figures 6H and 6I). From these results, we conclude that FAR2 plays an important role in very-long-chain FAl production in sebum.

AWAT2 produces WmEs and 2ω-WdiEs using FAls or FAl derivatives (1,ω-diols and/or diol-FA conjugates), respectively, and acyl-CoAs as substrates (Figure 7A).^18^ The production of meibum WmEs and 2ω-WdiEs, in which the FAs were almost exclusively long-chain FAs, was impaired in *Awat2* KO mice (Figure 8A).^18^ Although the quantities of WmEs and 2ω-WdiEs containing long-chain FAs in the sebum were also reduced in *Awat2* KO mice relative to WT mice, levels of those containing very-long-chain FAs were not (Figures 7D and 7G). These results indicate that AWAT2 shows substrate specificity toward long-chain acyl-CoAs, in agreement with an earlier *in vitro* study that examined this specificity.^22^ In that study, AWAT2 showed high activity toward saturated C10:0–C16:0 acyl-CoAs, low activity toward C18:0 acyl-CoA, and no activity toward C20:0 acyl-CoA. For mono-unsaturated acyl-CoAs, only C16:1 and C18:1 acyl-CoAs were tested, and AWAT2 exhibited high activity toward both. AWAT2 possesses two transmembrane domains at its N-terminus, which have been shown to contribute to the substrate specificity for acyl-CoAs.^30^ At present, the acyltransferase involved in the production of WmEs and 2ω-WdiEs containing very-long-chain FAs is unknown. AWAT2 belongs to the DGAT2 (diacylglycerol acyltransferase 2) family.^23^ In this family, there are six other members in addition to AWAT2 in mammals: DGAT2, AWAT1, DGAT2L6, and MGAT1–3. Of these, AWAT1 has the highest sequence homology to AWAT2, yet it was not involved in the production of WmEs containing very-long-chain FAs (Figure S2). Therefore, other DGAT2 family members or non-family members may be responsible for the production of these WmEs.

In this study, we revealed the detailed composition of WmEs and WdiEs and the presence of Chol-OAHFAs in mouse sebum. We also revealed the involvement of FAR2 and AWAT2 in the production of WEs containing very-long-chain FAls and long-chain FAs, respectively. These findings contribute to the elucidation of the molecular mechanisms behind the production of diverse WEs.

### Limitations of the study

In this study, we adopted a semi-quantitative approach to lipid profiling that prioritized broad coverage over strict quantification, particularly for lipid classes other than WmEs and Chol-Es, for which authentic standards are not available. However, since all species within each lipid class were analyzed under identical conditions, we believe that relative comparisons within each class are valid and biologically meaningful.

Our LC-MS/MS method provided information on carbon chain length and degree of unsaturation, but could not determine double-bond positions and offered only partial insight into chain branching. We observed two peaks with different retention times for several lipid classes (Figure S1). For WmE species containing saturated FAls, the earlier peak corresponds to species with *iso*- or *anteiso*-branched FAls, whereas the later peak corresponds to those with straight chains, based on our previous report on meibum lipids.^31^ However, under our LC conditions, it is not possible to distinguish whether the branching is *iso* or *anteiso*. To achieve more precise separation of straight-chain and branched-chain lipids and to discriminate between *iso*- and *anteiso*-branching, techniques such as electron ionization gas chromatography-MS or ion mobility-MS are preferable.^9^^,^^32^ For unsaturated lipid species, retention time is affected not only by the presence or absence of branching but also by the position and number of double bonds, making peak assignment difficult. Determining double bond positions requires techniques such as ozonolysis or the Paternò-Büchi reaction, followed by MS, with ion mobility separation providing improved resolution of positional isomers.^19^^,^^33^

The role of Chol-OAHFAs in sebum is still unclear. The acyltransferases involved in the production of WEs containing very-long-chain FAs remain to be identified, and the relationship between each WE class/species and sebum-related diseases, such as acne, seborrheic dermatitis, and alopecia, is unknown. These questions should be addressed in future research.

## Resource availability

### Lead contact

Further information and requests for resources should be directed to and, where possible, will be fulfilled by the Lead Contact, Akio Kihara (kihara@pharm.hokudai.ac.jp).

### Materials availability

All reagents used in this study will be made available on reasonable request to the lead contact.

### Data and code availability

• All data supporting the findings of this study are available within the article and its supplemental information or from the lead contact upon reasonable request.
• No original code was generated for this study.

## Acknowledgments

This work was supported by the 10.13039/501100001691Japan Society for the Promotion of Science (JSPS) 10.13039/501100001691KAKENHI grant numbers JP25K22534 and JP22H04986 (to A.K.).

## Author contributions

K.K., M.Y., and M.T. performed the experiments. K.J. supervised the project. A.K. planned and supervised the project and wrote the article.

## Declaration of interests

The authors declare no competing interests.

## Declaration of generative AI and AI-assisted technologies in the writing process

During the preparation of this work, the author used Microsoft M365 Copilot (GPT-5 chat model) in part to improve the readability and language of the article. After using this tool, the author reviewed and edited the content as needed and takes full responsibility for the final version of the article.

## STAR★Methods

### Key resources table

**Table:** 

REAGENT or RESOURCE	SOURCE	IDENTIFIER
**Chemicals, peptides, and recombinant proteins**		

C16:0 Cholesteryl-*d*_7_ ester	Avanti Research	Cat#700149
Behenyl oleate	Merck	Cat#O3255
Behenyl stearate	Nu-Chek Prep	Cat#WE-1333
Lauryl stearate	Larodan	Cat#45-1812
Lauryl oleate	Larodan	Cat#45-3812

**Experimental models: Organisms/strains**		

Mouse: C57BL/6J	Japan SLC	N/A
Mouse: *Awat2* KO	Sawai et al.^18^	N/A
Mouse: *Far2* KO	Otsuka et al.^21^	N/A

**Software and algorithms**		

MassLynx software	Waters	N/A
GraphPad Prism 10	GraphPad Software	N/A
IBM SPSS Statistics	IBM	N/A

**Other**		

PicoLab Rodent Diet 20	LabDiet	Cat#5053
Millex-LG syringe filter	Merck	Cat#SLLG025SS
Glass HPTLC Silica Gel 60	Merck	Cat#105641
Stereomicroscope Stemi DV4	Carl Zeiss	N/A
Zirconia beads	TOMY Seiko	Cat#ZB-10
Micro Smash MS-100R	TOMY Seiko	N/A
Xevo TQ-S LC–coupled triple quadrupole mass spectrometer	Waters	N/A
Xevo TQ-XS LC–coupled triple quadrupole mass spectrometer	Waters	N/A
ACQUITY UPLC CSH C18 Column	Waters	Cat#176002141

### Experimental model and study participant details

#### Mice

All mice used were male and of the C57BL/6J background. C57BL/6J mice were purchased from Japan SLC (Hamamatsu, Japan). *Far2* KO and *Awat*2 KO mice were previously described.^18^^,^^21^ Mice were housed in a specific pathogen-free environment under conditions of constant room temperature (23 ± 1°C), humidity (50 ± 5%), and a 12-h light/dark cycle. Water and food of standard chow diet (PicoLab Rodent Diet 20; LabDiet, St. Louis, MO, USA) were available *ad libitum*. Most experiments were performed using mice at 5–10 weeks of age, and mice at 6 or 9 months of age were used exclusively for photographic assessment of hair loss. For experiments involving different genotypes, mice of each genotype were transferred to separate cages at five weeks of age and housed separately for three weeks. The animal experiments were approved (No. 22–0036) by the institutional animal care and use committee of Hokkaido University.

### Method details

#### Lipid extraction

Lipids for measurements of WmEs in sebum were prepared as follows. Mouse back hairs (10 mg) were incubated with 600 μL of chloroform at room temperature for 1 h for lipid extraction and the supernatant was collected. Lipid extraction was repeated by adding 600 μL of methanol to the hair. After incubation for 1 h, the supernatant was collected and mixed with the previous one. The combined samples were then mixed with 540 μL of water, followed by phase separation by centrifugation (2,450 × *g*, room temperature, 3 min). The lower layer (organic phase) was collected and dried. The lipids obtained were suspended in 20 μL of chloroform/methanol (1:2, v/v) and separated via TLC using high-performance TLC plates (Glass HPTLC Silica Gel 60, Merck, Darmstadt, Germany) and hexane/toluene (1:1, v/v) as the resolving solvent. The silica containing WmEs was scraped off and incubated with 300 μL of ethanol at 37°C for 1 h for elution. After centrifugation (2,450 × *g*, room temperature, 3 min), the supernatant was collected. The elution step was repeated, and the supernatant was combined with the previous one and dried. To remove the silica completely, the samples were suspended in 200 μL of hexane, mixed with 200 μL of water, and separated into two phases via centrifugation (2,450 × *g*, room temperature, 3 min). The upper layer (organic phase) was collected, dried, suspended in 50 μL or 100 μL of chloroform/methanol (1:2, v/v), and subjected to LC-MS/MS analyses as described below.

Lipids for the measurement of WdiEs, Chol-OAHFAs, and Chol-Es in sebum were prepared as follows. Mouse back hairs (10 mg) were incubated with 600 μL of chloroform and the 250 pmol C16:0 Chol-E internal standard labeled with seven deuteriums (C16:0 Chol-E-*d*_7_; Avanti Research, Alabaster, AL, USA) at room temperature for 1 h, and the supernatant was collected. Lipid extraction was repeated as described above using 600 μL of methanol. To remove hairs, the samples were passed through a filter (Millex-LG; pore size, 0.20 μm; Merck). The lipids remaining on the filter were completely eluted with 1,200 μL of chloroform/methanol (1:1, v/v) and combined with the flow-through fraction. The samples were then mixed with 1,080 μL of water, and phases were separated by centrifugation (2,450 × *g*, room temperature, 3 min). The lower layer (organic phase) was collected and dried. To further remove water-soluble components, the samples were suspended in 200 μL of hexane, mixed with 200 μL of water, and subjected to phase separation via centrifugation (2,450 × *g*, room temperature, 3 min). The upper layer (organic phase) was collected, dried, suspended in 100 μL of chloroform/methanol (1:2, v/v), diluted with chloroform/methanol (1:2, v/v) to an appropriate concentration (Chol-Es, 50-fold; WdiEs and Chol-OAHFAs, 10-fold), and subjected to LC-MS/MS analyses as described below.

Lipids for the measurements of WmEs in meibum were prepared as follows. After the mice were euthanized, the upper and lower eyelids were excised under a stereomicroscope (Stemi DV4; Carl Zeiss, Oberkochen, Germany) and transferred to tubes containing zirconia beads (TOMY Seiko, Tokyo, Japan). The samples were mixed with 600 μL of chloroform/methanol (1:2, v/v) and the internal standard lauryl stearate (1 nmol C18:0 FA/C12:0 FAl WmE; Larodan, Solna, Sweden) and lauryl oleate (1 nmol C18:1 FA/C12:0 FAl WmE; Larodan) and homogenized using a Micro Smash MS-100R (TOMY Seiko) at 4,500 rpm and 4°C for 1 min. After centrifugation (20,400 × *g*, 4°C, 3 min), the supernatant was collected. The remaining pellet was re-extracted by adding another 600 μL of chloroform/methanol (1:2, v/v), followed by homogenization and centrifugation. The two supernatants were combined, mixed with 400 μL of chloroform and 720 μL of water for phase separation, and centrifuged (2,450 × *g*, room temperature, 3 min). The organic phase was collected and dried. WmEs were purified via TLC and hexane/methanol phase separation and then subjected to LC-MS/MS, as described previously.^18^

#### LC-MS/MS analyses

Ultra-performance LC-equipped triple quadrupole mass spectrometers (Xevo TQ-S and Xevo TQ-XS; Waters, Milford, MA, USA) were used for LC-MS/MS analyses. A reversed-phase column (ACQUITY UPLC CSH C18 column; particle size 1.7 μm, diameter 2.1 mm, length 100 mm; Waters) was used for LC separation. Lipids were separated at a flow rate of 0.3 mL/min using mobile phase A (acetonitrile/water [3:2, v/v] containing 5 mM ammonium formate) and mobile phase B (2-propanol/acetonitrile [9:1, v/v] containing 5 mM ammonium formate) with the gradient steps as follows: 0 min, 60% B; 0–21 min, gradient to 100% B; 21–25 min, 100% B; 25–30 min, gradient to 60% B. Lipids (injection volume, 5 μL) were ionized via electrospray ionization, and positive ions were detected via MS/MS.

For MS/MS analysis, two modes were employed: precursor ion scan and MRM. The ion source parameters were as follows: the capillary voltage was set to 3 kV for WmEs and WdiEs, 3.5 kV for Chol-OAHFAs, and 1.5 kV for Chol-Es. The cone voltage was set to 40 V for WmEs, 35 V for WdiEs and Chol-OAHFAs, and 15 V for Chol-Es. The desolvation temperature was 500°C for WmEs containing saturated FAs and Chol-OAHFAs, 650°C for WmEs containing mono-unsaturated FAs and WdiEs, and 450°C for Chol-Es. The desolvation gas flow was maintained at 1,200 L/h for all lipid classes. The source temperature was 150°C for WmEs, WdiEs, and Chol-OAHFAs, and 110°C for Chol-Es.

In precursor ion scan mode, the measured species and corresponding collision energies were as follows: Chol-OAHFA (*m/z* 1,184, 15 eV); 2α-WdiE (*m/z* 844, 30 eV); and 2ω-WdiE (*m/z* 1,094, 30 eV). In MRM mode, proton adducts were detected for both the precursor and product ions of WmEs, WdiEs, and Chol-OAHFAs. In contrast, ammonium adducts and proton adducts were detected for the precursor ions and product ions of Chol-Es, respectively, as described previously.^34^ The collision energies and *m/z* values of the precursor (Q1) and product ions (Q3) for WmEs, WdiEs, Chol-OAHFAs, and Chol-Es are shown in Tables S1, S2, S3, and S4. Note that WmEs containing saturated and mono-unsaturated FAs produce different types of product ions upon collision-induced dissociation in Q2.^35^ The dwell time was set to 0.03 s for all lipid classes. To quantify WmEs containing saturated FAs and mono-unsaturated FAs in sebum, behenyl stearate (C18:0 FA/C22:0 FAl WmE; Nu-Chek Prep, Elysian, MN, USA) and behenyl oleate (C18:1 FA/C22:0 FAl WmE, Merck) were used as external standards, respectively. Ten-point calibration curves were generated for each external standard, and samples were appropriately diluted to fall within the linear range for quantification. To quantify the Chol-Es in sebum and saturated or mono-unsaturated FA-containing WmEs in meibum, C16:0 Chol-E-*d*_7_, lauryl stearate, and lauryl oleate were used as internal standards, respectively. Each internal standard was tested at three different amounts during preliminary experiments, and the most suitable amount was selected for quantification. MassLynx software (Waters) was used for data analysis.

#### Real-time quantitative RT-PCR

Skin preparation and subsequent quantitative real-time RT-PCR were performed as previously described,^36^ except for omission of the RNA purification step using a column. The primers used were identical to those reported earlier.^18^^,^^37^

### Quantification and statistical analysis

#### Statistics

Data are presented as means +standard deviation (SD). We used Prism version 10 (GraphPad Software, Boston, MA, USA) for Welch’s *t* test and IBM SPSS Statistics (IBM, New York, NY, USA) for Tukey’s test. Significance was defined as *p* < 0.05.

## Supplemental information

Document S1. Figures S1–S2 and Tables S3 and S4

Table S1. LC-MS/MS precursor and product ion m/z values and collision energies for WmEs, related to Figures 2 and 6–8

Table S2. LC-MS/MS precursor and product ion m/z values and collision energies for WdiEs, related to Figures 3, 4, and 6–8
