# Supplementary material for: Detailed composition of wax esters in mouse sebum and the involvement of FAR2 and AWAT2
Source: iScience. 2026 Jan 29;29(2):114836. doi: 10.1016/j.isci.2026.114836 (PMC12915242; doi:10.1016/j.isci.2026.114836)
Supplement: Document S1. Figures S1–S2 and Tables S3 and S4 [file mmc1.pdf]

iScience, Volume *29*

Supplemental information

Detailed composition of wax esters in mouse sebum and the involvement of FAR2 and AWAT2

Karin Kuribayashi, Keisuke Jojima, Moe Yamamoto, Mirei Takeda, and Akio Kihara

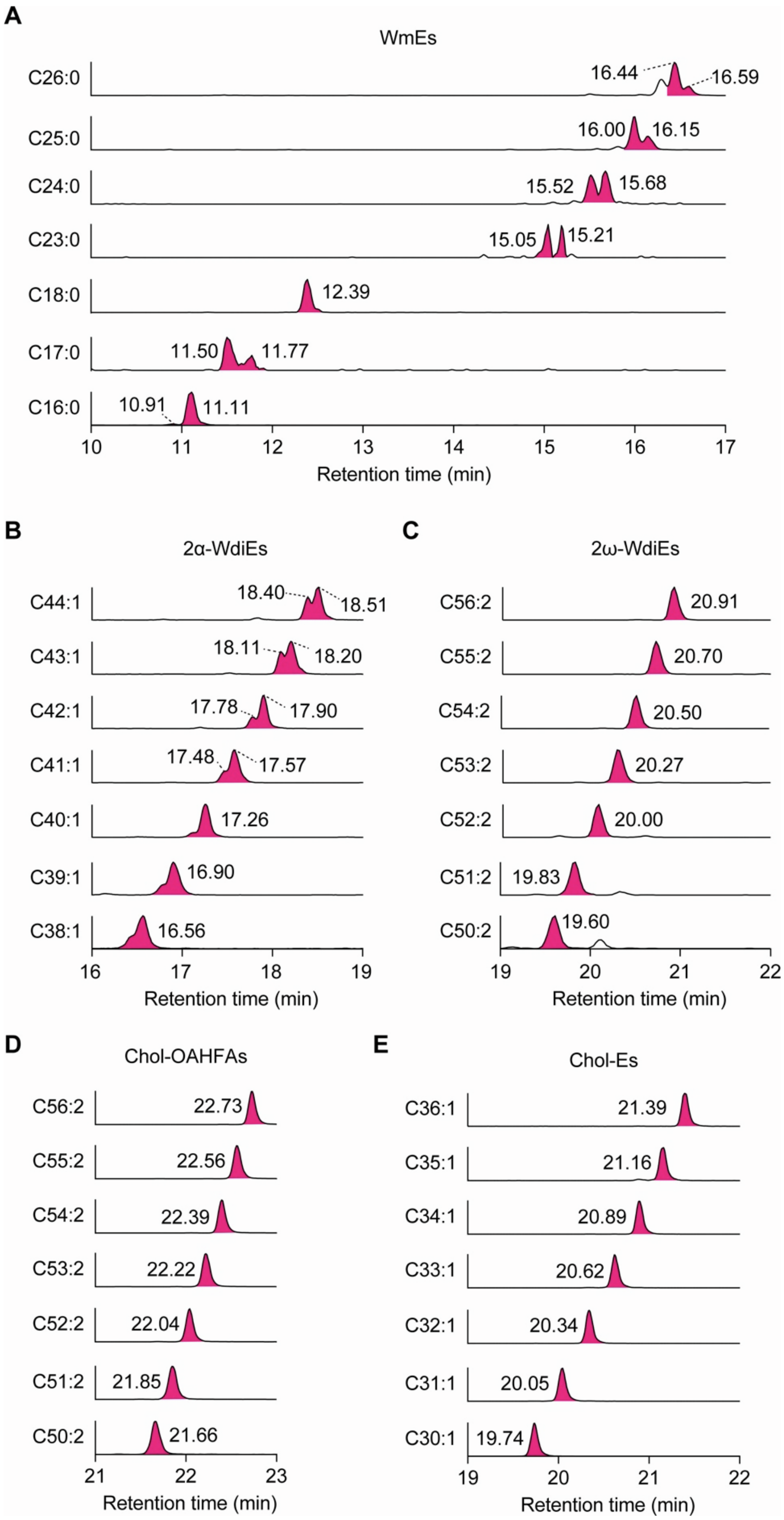

Figure S1. Chromatograms of WmEs, 2α-WdiEs, 2ω-WdiEs, Chol-OHAFAs, and Chol-Es. Related to Figures 2–5.

Lipids were extracted from the hair of 8-week-old male C57BL/6 mice and analyzed via LC-MS/MS in MRM mode to quantify WmEs (A), 2α-WdiEs (B), 2ω-WdiEs (C), Chol-OHAFAs (D), and Chol-Es

(E). Each panel represents the chromatogram of a representative species for each lipid class, as follows: WmEs containing C16:0 FA and the indicated saturated FAl; 2α-WdiEs containing C16:1 FA and the indicated mono-unsaturated diol-FA; 2ω-WdiEs containing C20:1 FA and the indicated di-unsaturated diol-FA; Chol-OHAFAs containing the indicated di-unsaturated OAHFA; and Chol-Es containing the indicated mono-unsaturated FA.

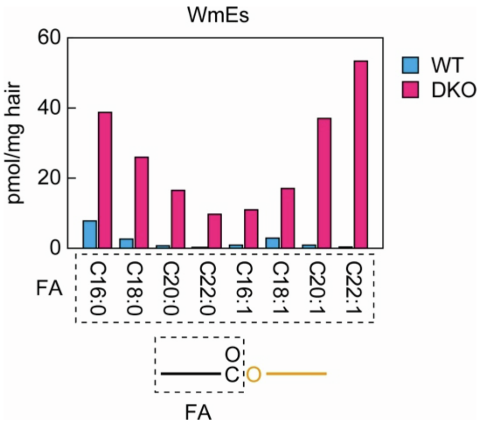

Figure S2. AWAT1 is not involved in WmE production in mouse sebum. Related to Figure 7.
WT and *Awat1 Awat2* double KO (DKO) mice (n = 1 each) at 8 weeks of age were transferred to separate cages and housed separately for 3 weeks. Lipids were extracted from the hair, and WmEs were analyzed via LC-MS/MS. Values are the mean +SD of the quantities of the WmEs containing the indicated FA moieties. The simplified structure of the WmE with the analyzed FA moiety is shown.

Table S3. LC-MS/MS precursor and product ion *m/z* values and collision energies for Chol-Es. Related to Figures 5–7.

FA	Precursor ion (Q1) $[M+NH_4]^+$	Product ion (Q3) $[Chol-H_2O]^+$	Collision energy (eV)
C16:0	642.5	369.4	15
C17:0	656.5	369.4	15
C18:0	670.5	369.4	15
C19:0	684.5	369.4	15
C20:0	698.6	369.4	15
C21:0	712.6	369.4	15
C22:0	726.6	369.4	15
C23:0	740.6	369.4	15
C24:0	754.6	369.4	15
C25:0	768.6	369.4	15
C26:0	782.6	369.4	15
C27:0	796.7	369.4	15
C28:0	810.7	369.4	15
C29:0	824.7	369.4	15
C30:0	838.7	369.4	15
C31:0	852.7	369.4	15
C32:0	866.7	369.4	15
C33:0	880.7	369.4	15
C34:0	894.8	369.4	15
C35:0	908.8	369.4	15
C36:0	922.8	369.4	15
C37:0	936.8	369.4	15
C38:0	950.8	369.4	15
C39:0	964.8	369.4	15
C40:0	978.8	369.4	15
C16:1	640.5	369.4	15
C17:1	654.5	369.4	15
C18:1	668.5	369.4	15
C19:1	682.5	369.4	15
C20:1	696.5	369.4	15
C21:1	710.6	369.4	15
C22:1	724.6	369.4	15
C23:1	738.6	369.4	15
C24:1	752.6	369.4	15

C25:1	766.6	369.4	15
C26:1	780.6	369.4	15
C27:1	794.6	369.4	15
C28:1	808.7	369.4	15
C29:1	822.7	369.4	15
C30:1	836.7	369.4	15
C31:1	850.7	369.4	15
C32:1	864.7	369.4	15
C33:1	878.7	369.4	15
C34:1	892.7	369.4	15
C35:1	906.8	369.4	15
C36:1	920.8	369.4	15
C37:1	934.8	369.4	15
C38:1	948.8	369.4	15
C39:1	962.8	369.4	15
C40:1	976.8	369.4	15
C16:2	638.5	369.4	15
C17:2	652.5	369.4	15
C18:2	666.5	369.4	15
C19:2	680.5	369.4	15
C20:2	694.5	369.4	15
C21:2	708.5	369.4	15
C22:2	722.6	369.4	15
C23:2	736.6	369.4	15
C24:2	750.6	369.4	15
C25:2	764.6	369.4	15
C26:2	778.6	369.4	15
C27:2	792.6	369.4	15
C28:2	806.6	369.4	15
C29:2	820.7	369.4	15
C30:2	834.7	369.4	15
C31:2	848.7	369.4	15
C32:2	862.7	369.4	15
C33:2	876.7	369.4	15
C34:2	890.7	369.4	15
C35:2	904.7	369.4	15
C36:2	918.8	369.4	15
C37:2	932.8	369.4	15

C38:2	946.8	369.4	15
C39:2	960.8	369.4	15
C40:2	974.8	369.4	15
d_7-C16:0	649.5	376.4	15

Table S4. LC-MS/MS precursor and product ion *m/z* values and collision energies for Chol-OAHFAs. Related to Figures 5–7.

OAHFA	Precursor ion (Q1) $[M + H]^+$	Product ion (Q3) $[Chol - H_2O]^+$	Collision energy (eV)
C46:1	1074.1	369.4	15
C47:1	1088.1	369.4	15
C48:1	1102.1	369.4	15
C49:1	1116.1	369.4	15
C50:1	1130.1	369.4	15
C51:1	1144.1	369.4	15
C52:1	1158.2	369.4	15
C53:1	1172.2	369.4	15
C54:1	1186.2	369.4	15
C55:1	1200.2	369.4	20
C56:1	1214.3	369.4	20
C57:1	1228.3	369.4	20
C58:1	1242.3	369.4	20
C59:1	1256.3	369.4	20
C60:1	1270.3	369.4	20
C61:1	1284.3	369.4	20
C62:1	1298.3	369.4	20
C63:1	1312.4	369.4	20
C64:1	1326.4	369.4	20
C65:1	1340.4	369.4	20
C66:1	1354.4	369.4	20
C46:2	1072.1	369.4	15
C47:2	1086.1	369.4	15
C48:2	1100.1	369.4	15
C49:2	1114.1	369.4	15
C50:2	1128.1	369.4	15
C51:2	1142.1	369.4	15
C52:2	1156.2	369.4	15
C53:2	1170.2	369.4	15
C54:2	1184.2	369.4	15
C55:2	1198.2	369.4	20
C56:2	1212.3	369.4	20
C57:2	1226.3	369.4	20
C58:2	1240.3	369.4	20

C59:2	1254.3	369.4	20
C60:2	1268.3	369.4	20
C61:2	1282.3	369.4	20
C62:2	1296.3	369.4	20
C63:2	1310.3	369.4	20
C64:2	1324.4	369.4	20
C65:2	1338.4	369.4	20
C66:2	1352.4	369.4	20
C46:3	1070.1	369.4	15
C47:3	1084.1	369.4	15
C48:3	1098.1	369.4	15
C49:3	1112.1	369.4	15
C50:3	1126.1	369.4	15
C51:3	1140.1	369.4	15
C52:3	1154.1	369.4	15
C53:3	1168.2	369.4	15
C54:3	1182.2	369.4	15
C55:3	1196.2	369.4	20
C56:3	1210.2	369.4	20
C57:3	1224.3	369.4	20
C58:3	1238.3	369.4	20
C59:3	1252.3	369.4	20
C60:3	1266.3	369.4	20
C61:3	1280.3	369.4	20
C62:3	1294.3	369.4	20
C63:3	1308.3	369.4	20
C64:3	1322.4	369.4	20
C65:3	1336.4	369.4	20
C66:3	1350.4	369.4	20
